# Randomised controlled trial using daily electrical muscle stimulation (EMS) in critically ill patients to prevent intensive care unit (icu) acquired weakness (ICUAW)

**DOI:** 10.1186/2197-425X-3-S1-A809

**Published:** 2015-10-01

**Authors:** M Goll, T Wollersheim, K Haas, R Moergeli, J Malleike, F Nehls, K Reiher, N Carbon, G Sonomoya, S Weber-Carstens

**Affiliations:** Charité - Universitätsmedizin Berlin, Berlin, Germany

## Introduction

Critically ill patients frequently suffer from intensive care unit (ICU) acquired weakness (ICUAW). Evoke muscle contraction by electrical muscle stimulation (EMS) may help to prevent their overall strength diminishing by conserving muscle strength and mass.

## Objectives

The aim of the study was to investigate the effects of daily EMS-therapy on muscular strength in ICU patients, compared to a protocol based standard physiotherapy.

## Methods

The interventional randomized controlled trial included critically ill patients at high risk for ICUAW selected by SOFA score ≥ 9 and onset of critical illness in less than 72 hours. Randomization was administered to EMS group (protocol base physiotherapy and additional EMS) or a physiotherapy group (protocol base physiotherapy). EMS-therapy was applied for 20 minutes 7 days/week to 8 bilateral muscle groups by an experienced physiotherapist. Medical-Research-Council (MRC) score for muscle strength and grip strength measured by hand-dynamometer were recorded on the first day patients were adequately awake and on the day of their ICU discharge. Non-parametric tests were performed. Ethic vote (Charité EA 2/041/10).

## Results

50 patients, 33 males and 17 females with a median age of 52 (10/90) years and a SOFA score of 13 (10/15) at admission where included in the study. Randomization resulted in 21 to 29 subjects for EMS and controls respectively. Time-points of muscle strength measurements were “first day being awake” at median day 11 (7/19) and discharge form ICU, median day 26 (17/33). Patient characteristics and time-points of recorded parameters were without significant differences between the intervention (EMS) and control group (data not shown). Overall subjects fulfilled criteria for ICUAW on their first day of being awake and significantly improved their MRC score and grip strength until their ICU discharge. Surprisingly we could not show direct group differences between EMS- and control group for MRC score and grip strength. By focusing separately on the course of strength for EMS- and control group we found an increase in MRC score for both groups between the first and second measurement.

Grip strength in nonEMS treated patients rose significantly whilst patients receiving EMS couldn't show significant improvements between measurements.

## Conclusions

Our patients managed to increase their grip- and general strength levels significantly from their first day of being adequately awake until the day of their ICU discharge. Further subgroup analysis however indicates that EMS therapy may not be more beneficial in increasing grip or MRC-measured strength compared to the standard physiotherapy protocol within our study. The missing grip strength improvement of the EMS group between the first day of being awake and ICU discharge may be attributed to greater muscle strength at time of awakening without significant power to show group differences.

## Grant Acknowledgment

DFG KFO 192/2 TP3

**Figure 1:**
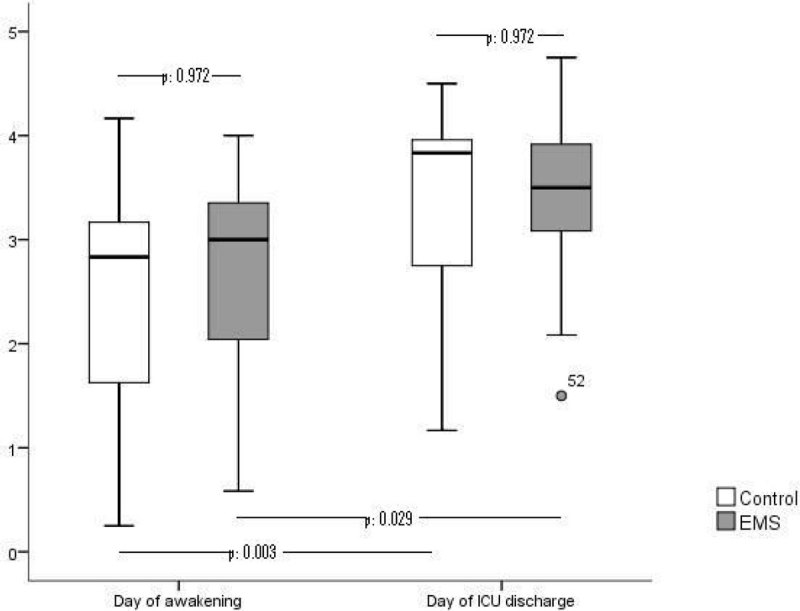
Figure 1

**Figure 2:**
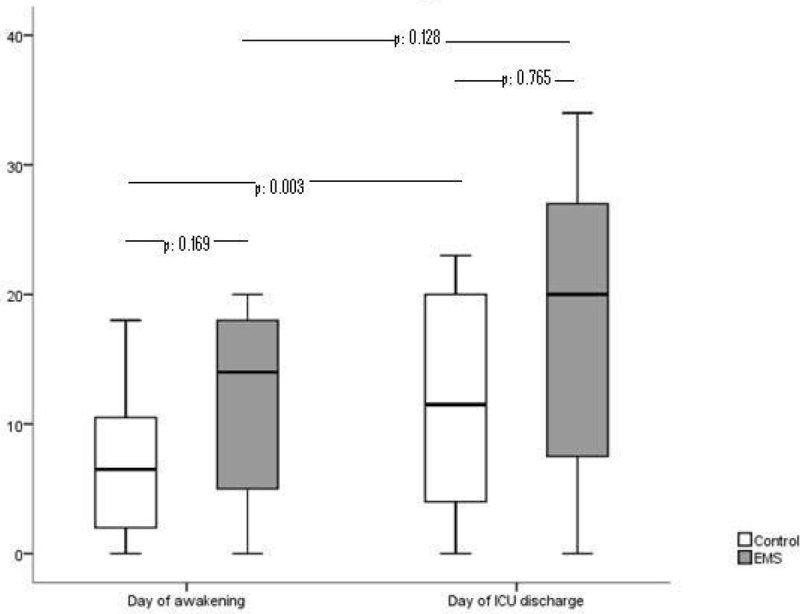
Figure 2

